# Saffold Cardioviruses of 3 Lineages in Children with Respiratory Tract Infections, Beijing, China

**DOI:** 10.3201/eid1607.091682

**Published:** 2010-07

**Authors:** Lili Ren, Richard Gonzalez, Zhengde Xie, Yan Xiao, Yongjun Li, Chunyan Liu, Lan Chen, Qingqing Yang, Guy Vernet, Gláucia Paranhos-Baccalà, Qi Jin, Kunling Shen, Jianwei Wang

**Affiliations:** Author affiliations: State Key Laboratory for Molecular Virology and Genetic Engineering, Beijing, People’s Republic of China (L. Ren, Q. Jin, J. Wang);; Institute of Pathogen Biology, Beijing (L. Ren, R. Gonzalez, Y. Xiao, L. Chen, Q. Yang, Q. Jin, J. Wang);; Fondation Mérieux, Lyon, France (R. Gonzalez, Y. Li, G. Vernet, G. Paranhos-Baccalà);; Beijing Children’s Hospital, Beijing (Z. Xie, C. Liu, K. Shen)

**Keywords:** Saffold cardiovirus, viruses, pediatric patients, respiratory tract infections, dispatch

## Abstract

To clarify the potential for respiratory transmission of Saffold cardiovirus (SAFV) and characterize the pathogen, we analyzed respiratory specimens from 1,558 pediatric patients in Beijing. We detected SAFV in 7 (0.5%) patients and identified lineages 1–3. However, because 3 patients had co-infections, we could not definitively say SAFV caused disease.

Saffold cardiovirus (SAFV) is a new piconavirus, originally identified from fecal samples of a female infant with fever of unknown origin (*1*). SAFV has since been reported worldwide, and 8 lineages have been identified (*1**–**6*). Although serologic surveys have shown that SAFV-3 infection occurs early in life (*7*), the pathogenicity of SAFV is still unclear.

Because SAFVs are mainly detected in fecal samples, virus transmission is thought to occur by the fecal–oral route (*1**,**3**–**7*). However, 2 research groups also found SAFV-2 lineage in respiratory secretions (*2**,**4*). Thus, we investigated whether the respiratory tract route could be an additional transmission route and whether SAFV lineages other than SAFV-2 may also infect the respiratory tract. We identified and characterized 7 SAFV strains, which belonged to 3 distinct lineages, from respiratory samples of children with lower and upper respiratory tract infections (LRTIs and URTIs, respectively).

## The Study

We assessed 2 cohorts. Cohort 1 comprised 1,032 children (617 boys and 415 girls) with acute LRTIs, hospitalized in Beijing Children’s Hospital (BCH), from whom nasopharyngeal aspirates were collected from May 2007 through March 2009. The patients ranged in age from 2 weeks to 16 years (mean age 31.3 months, median 9 months). Cohort 2 comprised 506 BCH outpatient children (277 boys and 229 girls) with acute URTIs, from whom throat swabs were collected from May through August 2009. These patients ranged in age from 4 months to 16 years (mean age 46.7 months, median 37 months).

Virus nucleic acids in clinical samples were extracted by using the NucliSens easyMAG system (bioMérieux, Marcy-l’Etoile, France) according to the manufacturer’s instructions. SAFV RNA was detected by nested reverse transcription–PCR (RT-PCR) by using primers selective for the 5′ untranslated region (UTR) (*3*). The viral protein (VP) 1 gene was amplified by using 3 pairs of primers as previously described (*3**,**5**,**6*). The full genomic sequences were obtained by a genome walking method (*7*). The 5′ and 3′ UTR sequences were determined by using the RACE System (Invitrogen, Carlsbad, CA, USA) according to the manufacturer’s protocol. After being cloned into the pGEM-T Easy vector (Promega, Madison, WI, USA), all PCR products were verified by sequencing. In addition, all screened specimens were tested for known respiratory viruses as previously described (*8**,**9*). *Mycoplasma pneumoniae* was detected by using the gelatin particle agglutination test kit (SERODIA-MYCO II, Fujirebio, Japan).

For phylogenetic analysis, we constructed neighbor-joining trees based on the distances of SAFV nucleotide or amino acid sequences by using MEGA 4.0 (*10*). We used SimPlot (version 3.5.1) to analyze possible recombination between viral genome sequences (*11*).

LLC-MK2 cells were used to isolate SAFV as previously described (*12*). Cells were collected either when cytopathic effects were observed or after 12 days postinoculation, and then they were tested for SAFV by RT-PCR.

We detected SAFV RNA in 4 (0.4%) of the 1,032 nasopharyngeal aspirates from patients with LRTIs and in 3 (0.6%) of the 506 throat swab specimens from outpatients with URTIs. The SAFV-positive patients (4 girls and 3 boys) were 5 months to 9 years of age (Table). SAFV infection did not appear to have a predominant time for occurrence: cases were detected in a range of months for the periods covered (August and December 2007, October and November 2008, and June 2009). All SAFV-positive patients exhibited symptoms of respiratory tract infection, such as coughing, gasping, sneezing, or fever. Of the 4 SAFV-positive patients with LRTIs, 2 had underlying illnesses, i.e., tuberculosis, hepatic dysfunction, or respiratory failure (Table). All SAFV-positive patients recovered within 7–11 days. No major differences were found in disease duration between patients who had underlying diseases and those who did not.

Co-infections with additional respiratory pathogens were detected for 3 of 7 SAFV-positive patients, all in the first cohort. These pathogens were respiratory syncytial virus (1 patient), enterovirus (1 patient), and *M. pneumoniae* (1 patient) (Table). A SAFV-3 strain was isolated from sample BCH1031. Starting at 5 days postinoculation, cytopathic effects were observed. Virus was not isolated from other SAFV-positive samples (data not shown).

**Figure 1:**
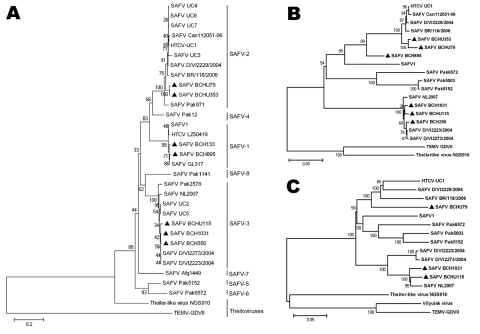
Phylogenetic analysis of Saffold cardiovirus (SAFV) strains obtained in Beijing, China, 2007–2009, based on viral protein (VP) 1 (A), P1 capsid proteins (B), and full-length genomes (C). The trees, with 500 bootstrap replicates, were generated by using the neighbor-joining algorithm in MEGA 4.0 (*10*). Strains identified in this study are indicated by a specific identification code (BCH or BCHU), followed by the patient number and labeled with dark triangles (BCH133, BCH350, BCH895, BCH1031, BCHU79, BCHU115, and BCHU353). GenBank accession nos. of the complete genome sequences are BCH1031 GU943513, BCHU115 GU943514, and BCHU79 GU943518; of the P1 gene, BCH350 GU943515, BCH895 GU943516, and BCHU353 GU943517; and of the VP1 gene of BCH133, GU126461. SAFV-1 (prototype), UC1-UC7, Can112051-06, D/VI2229/2004, BR/118/2006, D/VI2273/2004, D/VI2223/2004, LZ50419, GL317, Pak12, Pak971, Afg1449, Pak1141, Pak2578, Pak5003, Pak5152, Pak6572, and NL2007, TEMV-GDVII, Theiler-like virus NGS910, and Vilyuisk virus were used as reference sequences (GenBank accession nos. NC009448, NC010810, EU604745-EU604750, AM922293, EU681176-EU681179, FJ586240, FJ464767, FJ463600-FJ463602, FJ463604, FJ463605, FJ463615-FJ463617, FM207487, X56019, AB090161, and EU723237). Scale bars indicate nucleotide substitutions per site.

We identified 3 genetic lineages, SAFV-1, -2 and -3, on the basis of phylogenetic analysis of VP1, the most diverse protein of picornaviruses (*6*) (Figure 1). Multiple-alignment analysis, based on reference sequences of each lineage available in GenBank, showed that the identity of the VP1 amino acid sequences among the strains in the same lineage was 92.6%–100% and among strains belonging to different lineages, 63.3%–100% (Table A1). We found no obvious differences in amino acid sequences, nor any new motifs in these sequences, between strains detected in respiratory samples and those in fecal samples within the same lineage.

**Figure 2:**
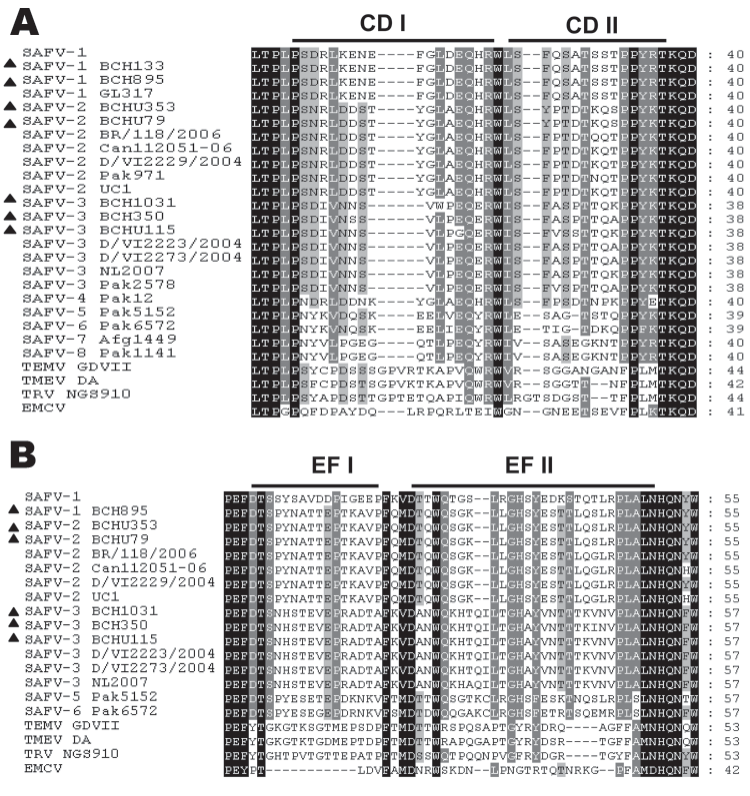
Alignment of Saffold cardiovirus (SAFV) viral protein (VP) 1 CD (A) and VP2 EF (B) loop sequences from strains isolated in Beijing, China, 2007–2009. Columns highlighted in black show absolute amino acid conservation; those highlighted in gray show amino acids with highly similar properties. Strains identified in this study are labeled with dark triangles (BCH133, BCH1031, BCHU115, BCH350, BCH895, BCHU353 and BCHU79) (GenBank accession nos. GU126461, GU943513–GU943518), SAFV-1 (prototype), SAFV-1 GL317, SAFV-2 Can112051-06, SAFV-2 BR/118/2006, SAFV-2 D/VI/2229/2004, SAFV-2 Pak971, SAFV-2 UC1, SAFV-3 Pak2578, SAFV-3 D/VI/2223/2004, SAFV-3 D/VI/2273/2004, SAFV-3 NL2007, SAFV-4 Pak12, SAFV-5 Pak5152, SAFV-6 Pak 6572, SAFV-7 Afg1449, SAFV-8 Pak1141, Theiler-like virus NGS910, TMEV GDVII, and EMCV were used as reference sequences (GenBank accession nos. NC009448, FJ464767, AM922293, EU681177, EU681176, FJ463601, NC010810, FJ463605, EU681179, EU681178, FM207487, FJ463600, FJ463616, FJ463617, FJ463602, FJ463604, AB090161, X56019, X87335).

To further characterize the variation of SAFVs, we amplified the P1 region sequences of 6 strains identified in this study. The identity of all available nucleotide sequences of the P1 region among SAFVs was 68.5%–97.2%, whereas that of amino acid sequences was 74.9%–99.4%. The VP1 CD and VP2 EF loop structures, which display the greatest amino acid divergence among different SAFVs and are associated with tropism and virulence (*6**,**7*), were analyzed by amino acid alignment. Similar to previously reported findings (*6**,**7*), we found that the sequences in both loops among SAFVs were highly diverse among SAFVs (Figure 2). The amino acid identities among all known SAFVs were 53.1%–100% in CD loops and 40.3%–100% in EF loops; amino acid identities of SAFVs versus animal cardioviruses were 29.1%–41.1% in CD loops and 19.2%–32.6% in EF loops. We did not find any major differences among the available amino acid sequences of CD loops (91.1%–100.0%) or EF loops (97.9%–100.0%) of SAFVs within the same lineage for samples collected from either the gastrointestinal or respiratory tracts.

**Figure A1:**
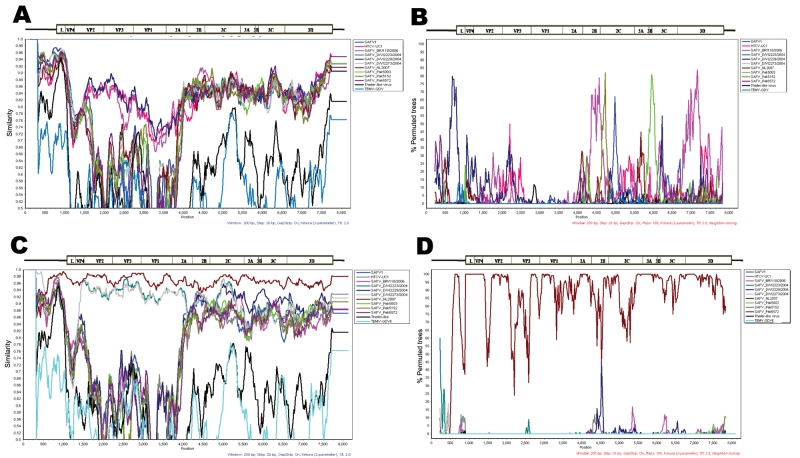
Recombination analyses of Saffold cardioviuses (SAFVs), isolated in Beijing, China, 2007–2009. The relationships among the aligned cardiovirus genome sequences were analyzed by SimPlot software version 3.5.1 (*11*). A) Sliding-window SimPlot graph generated by using the sequence of the BCHU79 (SAFV-2) as a query against SAFVs, Theiler-like virus, and Theiler murine encephalomyelitis virus–GDVII. B) Bootscan analysis of the sequence of SAFV BCHU79 in comparison to SAFVs, Theiler-like virus, and Theiler murine encephalomyelitis virus–GDVII. C) Sliding-window SimPlot graph generated by using the sequence of the BCH1031 (SAFV-3) as a query against SAFVs, Theiler-like virus, and Theiler murine encephalomyelitis virus–GDVII. D) Bootscan analysis of the sequence of SAFV BCH1031 in comparison to SAFVs, Theiler-like virus, and Theiler murine encephalomyelitis virus–GDVII. Because the genome sequence identity between BCH1031 and BCH115 is as high as 94%, BCH1031 was used to conduct the SimPlot analysis. The nucleotide position is given on the x-axis, and the percent nucleotide sequence similarity is given on the y-axis. The positions relative to viral genes are shown by genome diagrams above the plots and scans. SAFV-1 (prototype), SAFV-2 UC1, SAFV-2 D/VI/2229/2004, SAFV-2 BR118/2006, SAFV-3 D/VI/2273/2004, SAFV-3 D/VI/2223/2004, SAFV-3 NL2007, SAFV-5 Pak5003, SAFV-5 Pak5152, SAFV-6 Pak 6572, Theiler-like virus NGS910, and Theiler murine encephalomyelitis virus–GDVII were used as reference sequences (GenBank accession nos. NC009448, NC010810, EU681176–EU681179, FM207487, FJ463615–FJ463617, AB090161, and X56019).

The complete genome sequence of BCH1031 (SAFV-3) was obtained, as were nearly complete genome sequences of BCHU115 (SAFV-3) and BCHU79 (SAFV-2), which covered full-length coding sequences as well as the 3′ UTR and a partial 5′ UTR (Figure 1). SimPlot analysis showed relatively high (>86%) identity between strains within the same lineage. No clear evidence of genetic recombination between the SAFV strains was found (Figure A1).

## Conclusions

Although 8 lineages of SAFV have been detected in fecal samples worldwide, only SAFV-2 has been detected in respiratory samples (*2**,**4*). In addition, only SAFV-1 had been reported in China (*5**,**13*). In this study, we found that SAFV lineages 1, 2, and 3 co-circulated in patients with respiratory infections in Beijing.

Although SAFV is known to be transmitted by the fecal–oral route (*1**,**3**–**7*), as are other picornaviruses (*14*), our detection of SAFV in respiratory samples suggests that various SAFV lineages may also be transmitted through the respiratory tract and may be associated with disease. However, other respiratory viruses and *M. pneumoniae* were co-detected in 3 of the 7 SAFV-positive patients. Given that we did not conduct assays for common respiratory bacteria (and the number of SAFV-positive cases was limited), whether SAFV actually caused the observed symptoms in the patients cannot be definitively determined.

The genetic diversity of SAFVs in respiratory samples can complicate the relationship between SAFVs and disease because different genotypes of the same picornavirus species may cause different clinical signs and symptoms (*6**,**14*). Further investigations are needed to clarify any possible link between the pathogenicity and genetic diversity of SAFV.

## Appendix

**Table A1:** Viral protein 1 amino acid sequence identities between Saffold cardiovirus strains

Genotype and sample code	BCH133	BCH895	SAFV-1	GL317	BCHU79	BCHU353	UC4	D/VI2229	Can112051-06	Pak971	BCHU115	BCH350	BCH1031	D/VI2223	UC2	Pak2678	Pak12	Pak5152	Pak6572	Afg1449	Pak1141
SAFV-1																					
BCH133	100																				
BCH895	97.8	100																			
SAFV-1	97.4	98.1	100																		
GL317	98.5	99.2	98.9	100																	
SAFV-2																					
BCHU79	73.7	74.0	75.5	74.8	100																
BCHU353	73.7	74.0	75.5	74.8	99.2	100															
UC4	74.8	74.8	76.2	75.5	95.2	95.2	100														
D/VI2229	74.4	74.4	75.9	75.1	93.7	93.7	97.4	100													
Can112051-06	75.1	75.1	76.6	75.9	94.4	94.4	98.5	96.6	100												
Pak971	75.1	75.1	76.6	75.9	93.3	93.3	94.1	92.6	93.7	100											
SAFV-3																					
BCHU115	65.3	65.3	65.6	66.0	70.9	70.9	70.9	70.5	71.6	69.1	100										
BCH350	66.0	66.0	66.4	66.7	71.3	71.3	71.3	70.9	72.0	69.1	96.6	100									
BCH1031	66.0	66.0	66.4	66.7	71.3	71.3	71.3	70.9	72.0	69.1	97.4	98.5	100								
D/VI2223	66.4	66.4	66.7	67.1	71.6	71.6	71.6	71.3	72.4	69.4	97.4	99.2	99.2	100							
UC2	66.4	66.4	66.7	67.1	72.0	72.0	72.0	71.6	72.4	69.4	96.2	98.1	98.1	98.8	100						
Pak2578	65.6	65.6	66.0	66.4	71.6	71.6	70.9	70.9	71.6	70.2	94.8	95.9	95.9	96.6	96.2	100					
SAFV-4																					
Pak12	79.5	79.9	81.7	80.6	81.6	81.9	81.9	81.2	81.6	83.0	67.6	67.6	68.0	68.0	68.3	68.0	100				
SAFV-5																					
Pak5152	67.2	67.6	68.3	67.6	67.0	67.0	65.2	65.5	65.5	67.7	67.3	68.1	68.1	68.4	68.4	67.3	66.6	100			
SAFV-6																					
Pak6572	64.7	64.3	65.0	64.7	63.3	63.3	63.0	63.3	63.3	63.7	64.4	65.2	65.2	65.5	65.9	64.8	64.8	80.8	100		
SAFV-7																					
Afg1449	67.8	67.8	69.3	68.6	68.0	68.0	68.3	68.7	68.3	68.3	69.8	70.9	70.9	71.3	71.3	70.5	69.8	69.9	67.0	100	69.1
SAFV-8																					
Pak1141	64.5	64.5	64.9	65.3	64.3	64.3	65.4	65.4	65.8	67.6	75.5	75.1	75.1	75.5	75.5	74.8	66.1	69.9	67.0	69.1	100

*All results are based on the pairwise analysis of sequences of Saffold cardiovirus (SAFV) viral protein 1 genes by using MEGA4.0 (*10*). The final dataset contained a total of 274 positions. GenBank accession numbers are given in the legend of Figure 1.

**Table:** Characteristics of patients with Saffold cardiovirus, Beijing, China, 2007–2009*

Patient no.	Age/sex	Sampling date	Diagnosis	Clinical signs	Other underlying diseases	Co-infecting organisms	SAFV lineage
BCH133	9 y/F	2007 Aug 7	Pneumonia	Cough	Tuberculosis, hepatic dysfunction	*Mycoplasma pneumoniae*	1
BCH350	5 mo/M	2007 Dec 7	Broncho- pneumonia	Cough, gasping	Respiratory failure	RSV	3
BCH895	2 y, 8 mo/M	2008 Oct 6	Pneumonia	Fever, cough, spitting, gasping, chills	No	EV	1
BCH1031	4 y/M	2008 Nov 16	Peribronchitis	Fever, cough, spitting, gasping, chills, sneezing	No	None	3
BCHU79	1 y, 6 mo/F	2009 Jun 5	URTI	Fever, cough	Unknown	None	2
BCHU115	1 y, 10 mo/F	2009 Jun 6	URTI	Fever, cough	Unknown	None	3
BCHU353	4 y, 6 mo/F	2009 Jun 17	URTI	Fever, cough	Unknown	None	2

*SAFV, Saffold cardiovirus; RSV, respiratory syncytial virus; EV, enterovirus; URTI, upper respiratory tract infection.
